# The Australian Racism, Acceptance, and Cultural-Ethnocentrism Scale (RACES): item response theory findings

**DOI:** 10.1186/s12939-016-0338-4

**Published:** 2016-03-17

**Authors:** Kaine Grigg, Lenore Manderson

**Affiliations:** School of Psychological Sciences, Monash University, Building 17, Clayton Campus, Wellington Road, Melbourne, VIC 3800 Australia; School of Public Health, University of the Witwatersrand, Johannesburg, South Africa

**Keywords:** Australia, Racism, Scale, Item Response Theory, Rasch analysis

## Abstract

**Background:**

Racism and associated discrimination are pervasive and persistent challenges with multiple cumulative deleterious effects contributing to inequities in various health outcomes. Globally, research over the past decade has shown consistent associations between racism and negative health concerns. Such research confirms that race endures as one of the strongest predictors of poor health. Due to the lack of validated Australian measures of racist attitudes, RACES (Racism, Acceptance, and Cultural-Ethnocentrism Scale) was developed.

**Methods:**

Here, we examine RACES’ psychometric properties, including the latent structure, utilising Item Response Theory (IRT). Unidimensional and Multidimensional Rating Scale Model (RSM) Rasch analyses were utilised with 296 Victorian primary school students and 182 adolescents and 220 adults from the Australian community.

**Results:**

RACES was demonstrated to be a robust 24-item three-dimensional scale of Accepting Attitudes (12 items), Racist Attitudes (8 items), and Ethnocentric Attitudes (4 items). RSM Rasch analyses provide strong support for the instrument as a robust measure of racist attitudes in the Australian context, and for the overall factorial and construct validity of RACES across primary school children, adolescents, and adults.

**Conclusions:**

RACES provides a reliable and valid measure that can be utilised across the lifespan to evaluate attitudes towards all racial, ethnic, cultural, and religious groups. A core function of RACES is to assess the effectiveness of interventions to reduce community levels of racism and in turn inequities in health outcomes within Australia.

**Electronic supplementary material:**

The online version of this article (doi:10.1186/s12939-016-0338-4) contains supplementary material, which is available to authorized users.

## Background

Racism and associated discrimination are pervasive and persistent challenges that permeate contemporary society, with multiple cumulative deleterious effects on the health of all people. Research consistently confirms that race is one of the strongest predictors of health outcomes, with racism a fundamental cause of such inequalities [1, 2]. Positive social contact is essential for social, psychological, and physiological health and development throughout the lifespan; individuals who experience social isolation or rejection, including as a result of inter- and intra-racial racism, are susceptible to various behavioural, emotional, and physical problems, and negative educational, economic, and social outcomes [3, 4]. Racist attitudes result in poor physiological outcomes, negative mental health outcomes, and general psychopathology in various minority racial, ethnic, cultural, and religious groups in numerous societies with immigrant and Indigenous populations [5–7]. Racism is also a key influence on common psychiatric conditions such as mood, anxiety, and eating and substance use disorders. Moreover, when groups are relentlessly depicted as problematic and undesirable, these stereotypes are internalised, with negative consequences for both dominant and non-dominant groups (cf. [8–11]).

Although a change in one’s beliefs or attitudes toward a stereotyped group may or may not lead to changes in behaviour toward members of that group [12], attitude change is an essential component of reducing community levels of racism. Measurement is therefore fundamental in discussions of improving racial attitudes [13]. Quantifying racism is challenging, however, requiring differentiation of its multiple dimensions and the range of potential reactions and responses to exposure to racism.

## Measuring racism

Racism research has historically concentrated on two alternate and distinct methods of measurement. The majority of investigations have examined the effects of racism by concentrating on victims of perceived racism, and evaluating the frequency and intensity of racist events on individuals (for reviews see [5, 6, 14–17]). Less attention has been paid to racist attitudes held by individuals. Even so, over 100 instruments exist which assess explicit racist attitudes and 24 are available to evaluate perceived racism [14, 18]. Most of these have not been appropriately validated, the tools often fail to meet minimum standards required for scientific attitude scales (fewer than 5 % of studies address a sufficient range of reliability and validity indices for the instrument to be considered valid), and they are often used indiscriminately. In addition, most measures of racist attitudes relate to anti-African American attitudes and are validated only for US populations. These scales may not necessarily be relevant, generalisable, valid, or useful in alternate settings. Further, direct extrapolation of US experiences and research is inappropriate for the Australian context [19], given the distinctive histories and experiences of Aboriginal Australians and African Americans; nature of colonial relations; extensiveness of genocidal pasts; relative size of populations; level of visibility; and extent of reduced social, economic, and health status [20]. Dissimilar patterns of cultural diversity across the two countries also render problematic the direct transfer of US measures to Australia.

Despite these problems, Australian researchers have often uncritically imported and utilised US concepts and tools [19, 20]. Several Australian scales have been developed, but these either concentrate on a specific group (e.g., Indigenous Australians; [19]) or lack a robust research base and peer evaluation of their empirical development and validation (e.g., [21]). This gap is especially apparent for youth: here the available instruments are limited to measures of social distance and stereotyping (e.g., [22, 23]); those adapted from non-Australian measures used without further validation (e.g., [24]); and instruments requiring extrapolation from participant responses, raising questions of reliability and validity (e.g., [25]).

Moreover, Australian studies of racism have predominantly been conducted as if racism existed only between White non-Indigenous and Indigenous Australians [26], with the first systematic investigation of racist attitudes in a minority group conducted only recently [27]. This is problematic because of community diversity in Australia, the varying characterisations of non-Australians versus Australians [9], and evidence that distinct racial, ethnic, cultural, and religious groups experience and conceptualise racism in different ways [28–30, 31].

While early characterisations of Indigenous people provided the foundations for contemporary racist practices [26, 32], the contemporary context is important, given the changing nature of racism [33]. Pedersen, Clarke, Dudgeon, and Griffiths [34] describe the historical progression of racism in Australia as moving from targeting Yugoslavs, Italians, Asians, Arabs, to Afghans. The past decade would most appropriately also include people from the sub-continent of India and from Africa, both populations widely reported in the media as key out-groups in contemporary Australian society. The historical, contemporary, and regional factors that shape the different attitudes to these groups need to be understood and reflected in assessment instruments to ensure appropriate evaluation of interventions aiming to improve intergroup relations. Current racism research is therefore limited in terms of generalisability, validity, and utility for the Australian context [35].

### Racism, Acceptance, and Cultural-Ethnocentrism Scale (RACES)

Despite the extensive work of Australian researchers and community and government organisations working against racism, there are no empirically validated tools available to measure racism in the Australian context. As a result, anti-racism programs are rarely well evaluated. To redress this, an explicit measure of racial, ethnic, cultural, and religious acceptance – the Australian Racism, Acceptance, and Cultural-Ethnocentrism Scale (RACES; [36]) – was developed with children, adolescents, and adults from various racial, ethnic, cultural, and religious backgrounds.

From December 2011 to March 2012, a qualitative study was conducted among young Australians on their conceptualisations of and experiences with racism, to generate sufficient data to form the basis of a scale (detailed elsewhere; [31]). This study demonstrated a consistent explanatory model for understanding racism across groups [36]. The qualitative data, which provided insight into Australian lay understandings of racism [31], were supplemented and complemented by an extensive and comprehensive literature review on the conceptual racism literature and existing instruments, to create the preliminary measure. Since RACES was designed to evaluate and inform anti-racism and pro-diversity initiatives, items were designed to measure acceptance of difference and racism viewed along a continuum. Efforts were made to ensure that the development of the items was atheoretical, primarily driven by the qualitative data, rather than conforming to a chosen theory of racism. Consequently, the items developed can be thought of as representing the multidimensional nature of contemporary racism in Australia, spanning a number of theoretical positions.

The items underwent expert review for appropriateness, comprehensiveness, redundancy, and clarity, and were consequently pilot tested utilising cognitive interviewing techniques with children to ensure comprehensibility regardless of age. The instrument was evaluated longitudinally and cross-sectionally with school children, adolescents, and adults drawing upon Classical Testing Theory (CTT; [36]). As we illustrate below, estimates of internal consistency reliability,^1^ in addition to factorial,^2^ construct,^3^ convergent,^4^ and discriminant validity^5^ support the measure.

### Aim and hypotheses

In this article we examine the underlying factor structure of RACES using Item Response Theory (IRT) to further refine and finalise the measure developed using CTT. This provides additional support for its use as a robust tool to assess and evaluate racism reduction interventions. We hypothesised that the underlying factor structure of the measure would be consistent for CTT and IRT, and that the final measure would function comparably across children, adolescents, and adults.

## Method

### Research setting

The childhood component of the research was based in a small town, Greenfields (pseudonym), located in Cardinia Shire, approximately 55 km southeast from central Melbourne. The Shire, and the adjacent City of Casey, are among the most rapidly growing residential areas of Melbourne, with population estimates well exceeding projected growth forecasts of both the state of Victoria and the Australian nation [37–39]. The vast majority of inhabitants of Cardinia Shire, and their parents, are Australian-born, at rates much higher than the general state and national populations. However, this cultural uniformity will be substantially impacted by the projected increase in population, with increasing numbers of culturally and linguistically diverse migrants predicted [38]. The adolescent and adult components of the research were conducted throughout the Australian nation.

### Participants

The research reported here involved 296 students from the core Victorian study area. These students were enrolled in six different primary schools in years five or six. Two of the schools were government funded and secular, two were non-denominational Christian, one was Islamic, and one was Catholic. In addition, 402 community individuals aged 15 years or older also participated. Adolescents and adults from six of the seven Australian states and territories participated in the research (for details see: [36]). It was considered important to examine the children, adolescents, and adults separately due to differences in their general developmental stage [40–42] and the level of crystallisation of their racial attitudes [43]. Descriptive statistics for each sample are displayed in Tables 1 and 2 below.

**Table 1:** Descriptive statistics split by data set

		Total	Actual	Usable response rate	
Sample size	Primary school	296	213	72 %	
	15–20 years	182	147	81 %	
	Community	402	263	65 %	
		*M*	*SD*	Range	*N*
Age (Years)	Primary school	11.34	0.71	10–13	271
	15–20 Years	18.31	1.41	15–20	147
	Community	23.24	9.72	15–71	263
		Male	Female		*N*
Gender	Primary School	151 (56 %)	120 (44 %)		271
	15–20 years	46 (31 %)	101 (69 %)		147
	Community	71 (27 %)	192 (73 %)		263
		Australia	Other		*N*
Country of birth	Primary school	237 (87 %)	35 (13 %)		272
	15–20 years	91 (62 %)	56 (38 %)		147
	Community	182 (69 %)	81 (31 %)		263
			Australia	Other	*N*
Parent country of birth	Primary school	Mother	179 (67 %)	87 (33 %)	266
		Father	164 (62 %)	100 (38 %)	264
	15–20 years	Mother	73 (50 %)	74 (50 %)	147
		Father	68 (46 %)	79 (54 %)	
	Community	Mother	133 (51 %)	130 (49 %)	263
		Father	130 (49 %)	133 (51 %)	

*Note:* Various participants did not provide all requested demographic data; 15–20 Years sample is a subset of the Community sample

**Table 2:** Participant self-labelled racial/ethnic background descriptives split by data set

Racial/ethnic background	Primary school	15–20 years	Community	Total sample
Australian	129 (47 %)	50 (34 %)	99 (38 %)	228 (42 %)
Chinese	3 (1 %)	8 (5 %)	18 (7 %)	21 (4 %)
English	14 (5 %)	3 (2 %)	5 (2 %)	19 (4 %)
Anglo-Saxon	11 (4 %)	-	2 (1 %)	13 (2 %)
Indian	5 (2 %)	6 (4 %)	8 (3 %)	13 (2 %)
English/Australian	8 (3 %)	2 (1 %)	4 (2 %)	12 (2 %)
Afghani	10 (4 %)	1 (1 %)	1 (0 %)	11 (2 %)
Indigenous Australian	1 (0 %)	4 (3 %)	7 (3 %)	8 (1 %)
New Zealander	6 (2 %)	1 (1 %)	2 (1 %)	8 (1 %)
New Zealander/Australian	6 (2 %)	1 (1 %)	1 (0 %)	7 (1 %)
Vietnamese	-	3 (2 %)	7 (3 %)	7 (1 %)
Dutch	4 (1 %)	-	1 (0 %)	5 (1 %)
Indonesian	1 (0 %)	3 (2 %)	4 (2 %)	5 (1 %)
Sri Lankan	3 (1 %)	1 (1 %)	2 (1 %)	5 (1 %)
Chinese/Australian	-	3 (2 %)	4 (2 %)	4 (1 %)
Filipino	2 (1 %)	2 (1 %)	2 (1 %)	4 (1 %)
Greek/Australian	2 (1 %)	1 (1 %)	2 (1 %)	4 (1 %)
Other	67 (25 %)	58 (40 %)	94 (36 %)	179 (30 %)

*Note:* Other denotes racial/ethnic background not otherwise listed. Percentages may not sum to 100 % due to rounding

### Item response theory

The Rasch models originally proposed in the 1960s can be used to analyse categorical data from assessments designed to measure latent underlying variables such as abilities, attitudes, or personality traits [44]. Rasch models and the related Item Response Theory emphasise that the qualities of both the individual and the item influence item responses [45]. The core underlying theory is that there is a differential effect of item ‘difficulty’ on individuals at different trait levels [45]. For example, on a hypothetical measure of racist attitudes, of the two items “I hate people from other backgrounds” and “I have some minor racist tendencies,” the former is considerably more ‘difficult’ to endorse and would be expected to be sanctioned only by individuals high on the trait of racism. Conversely, the latter item may be endorsed by individuals who are much lower, as well as those moderate or high, on the trait of racism. Ratifying each item provides distinct information about individuals with differing levels of the underlying trait of racism. In contrast, CTT tends to treat each item as having the same ‘difficulty’ and ignores differing response patterns. This limits CTT in its ability to deal with an ordered continuum of items representing an underlying unidimensional construct and with summation of rating scale data [46]. Consequently, Rasch models and IRT can be utilised to perform advanced analytical techniques, which evaluate the differential effects of item ‘difficulty’ and individual trait level not otherwise available within a CTT framework.

In some instances, Rasch models and IRT have been considered psychometrically superior to CTT methods such as Principal Components Analysis, Exploratory Factor Analysis, Confirmatory Factor Analysis (CFA), and related statistical analyses, and appear to improve the precision and validity of psychological measurement [45, 47]. Both IRT and CTT methods have advantages and limitations, however, with certain statistical approaches more advantageous than others depending on the research purpose [48]. Moreover, there are underlying mathematical similarities between both methods [49]. Since neither has an overarching distinct advantage, the IRT and CTT were used interdependently to evaluate the psychometric properties of RACES [50].

### Procedure

#### Ethic, consent, and permissions

Ethics approval was received by Monash University Human Research Ethics Committee. Prior to participation, all participants were provided with the explanatory statement and given the option to decline involvement in the research.

#### Testing procedure

Initial instructions to participants outlined the purpose of the survey as inquiring about their thoughts and feelings towards people from the many different racial, ethnic, cultural, and religious backgrounds in Australia, with a number of examples of backgrounds provided (e.g., “Australian”, “Jewish”, “African”, etc.). Once the survey was completed, participants were thanked for their involvement in the research, but no post-testing feedback was provided.

#### Primary school data set procedure

The authors became involved with five participating schools when we were invited to evaluate the activities of a Victorian anti-racism program, known as “Building Harmony in the Cardinia Growth Corridor”. The principal of an additional school was approached directly by the authors for student participation to enable the inclusion and evaluation of attitudes of children not currently participating in an anti-racism and pro-diversity initiative. All schools obtained permission for students to participate from parents, with no parent declining their child’s participation.

All surveys were completed in September 2012 under the supervision of teachers during class. In five schools the survey was completed online (completion time 15–30 min); in the remaining school surveys were completed in hard copy (completion time 45–60 min). All responses were completed within 10 days of initiation of the survey, which included a demographic questionnaire, RACES [36], and the Strengths and Difficulties Questionnaire [51] (not analysed here). Data are referred to below as the ‘Primary School data set’.

#### Community data set procedure

Adolescent and adult community participants were recruited nationally via newspaper, radio, and online advertising. Participants aged 15 years or older were considered capable of providing informed consent for the purposes of the current research. Participants were able to access a link to the online survey or contact the authors directly to be provided with a web link or a hard copy survey via mail; all but four responses were completed online, between March 2012 and April 2013. The surveys took approximately 15 min to complete and included a demographic questionnaire, RACES [36], the Dunn and Geeraert [21] Racism Survey, and the Minnesota Temperament Inventory [52]; the latter two measures are not analysed here. Data from this group are labelled below the ‘Community data set’. Data were intended to be examined in entirety (‘Community data set’) and split by adolescents aged 15–20 years (‘15–20 years data set’) and adults aged 21 years and over (‘21+ years data set’) to explore the consistency of the measure across age groups. However, the 21+ years data set failed to meet minimum IRT assumptions and was omitted from independent analysis.

#### Data treatment

**Fig. 1:**
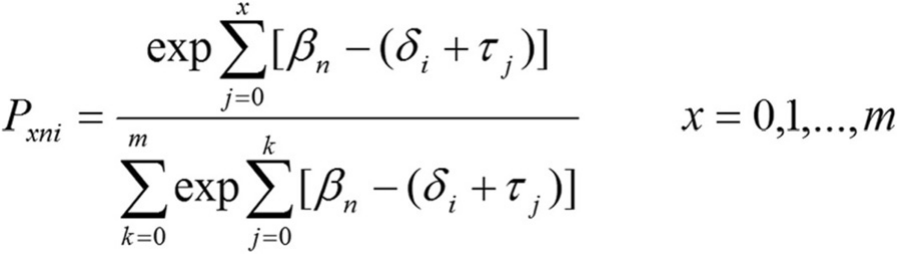
Rasch Rating Scale Model Part 1

**Fig. 2:**
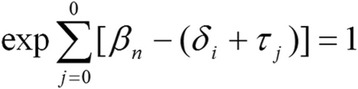
Rasch Rating Scale Model Part 2

Data for each data set – Primary School, Community, and 15–20 years – were initially collated in SPSS 20.0 and a missing data analysis was performed with all cases with 5 % or more data missing removed. Data were subsequently collated in ACER ConQuest 3.0. Analysis using a Rasch Rating Scale Model (RSM) was undertaken for each data set and each subscale separately. According to the model the probability of a person n responding in category x to item i, is given by Fig. 1:

where τ_ο_ = 0 so that Fig. 2:

β_n_ is the person’s position on the variable, δ_i_ is the scale value (‘difficulty’ to endorse) estimated for each item i and τ_1_, τ_2_, . . ., τ_m_ are the m response thresholds estimated for the m + 1 rating categories.

Model and item fit was assessed, and items were removed, according to criteria recommended by Linacre [53]. Infit (inlier-sensitive or information-weighted fit) and Outfit (outlier sensitive or non-weighted fit) were evaluated using 0.5–1.5 as a guideline for productive measurement, with values above 2.0 considered degrading of the measurement system. Standardised values, which assess if the model fits the data perfectly, were consequently inspected, allowing for−2.0–3.0 as an acceptable fit. Ill-fitting items on this index are not considered to be degrading of the overall model, but rather to be either overly predictable (i.e., > 3.0) or unpredictable (i.e., <−2.0). Moreover, if Infit and Outfit values are acceptable, Standardised values can be ignored [54]. Once misfitting items are identified, the researcher must make a decision to keep or disregard these data. The confirmation of item fit provides evidence of item quality and content validity.

### Measures

Both Primary School and Community participants completed the 25-item RACES, which consists of three subscales capturing a distinct component of racism: Racist Attitudes Scale (RAS), an 8-item scale of attitudes reflecting out-group denigration and derogation; Accepting Attitudes Scale (AAS), a 13-item scale of attitudes reflecting out-group endorsement and acceptance; and Ethnocentric Attitudes Scale (EAS), a 4-item scale of attitudes reflecting in-group favouritism and loyalty [36]. Items are responded to on a four-point Likert-type scale ranging from “Strongly Disagree” to “Strongly Agree”; half are reverse scored so higher scores indicate higher levels of acceptance or lower levels of racist attitudes. A neutral option was omitted to ensure ambivalent participants offered a meaningful response and to encourage them to consider their opinions when responding to the survey [55]. The subscales are appropriately interrelated with moderate to near perfect effect [36] and the relationships between RACES and an existing Australian measure of racism (very large to near perfect effect; [36]) and social, emotional, and behavioural strengths and difficulties (small to large effect; [56]) has been established. RACES has also been shown to be internally consistent (total scale and subscale Alpha Coefficient’s range from .79-.91); possesses factorial, construct, discriminant, and convergent validity in children, adolescents, and adults; and be test-retest reliable in children [36–57].

### Model selection

A core assumption of Rasch and IRT analyses is the selection of an appropriate model for the data [58]. A range of Rasch models can be utilised for rating scale type data; two competing models include the RSM and the Partial Credit Model (PCM). RSM specifies that a set of items share the same rating scale structure or response format (e.g., all items have the possible responses “Strongly Disagree”, “Disagree”, “Agree”, and “Strongly Agree”) [59, 60]. In contrast, PCM specifies that each item has its own unique rating scale structure, derived from assessments where responses that are incorrect can be indicative of some knowledge and are consequently given partial credit [59, 60]. For our purposes, a Rasch model known as a polytomous one parameter RSM for unidimensional traits was considered most appropriate [61]. The RSM was developed to analyse ratings from a unidimensional item set with two or more ordered and fixed response categories [62], and was expanded for use in multidimensional models in IRT software, such as ACER ConQuest 3.0. Both unidimensional and multidimensional RSM were utilised to examine the underlying latent structure as unidimensional (i.e., three unidimensional subscales examined independently) and multidimensional (i.e., three subscales examined interdependently as a single multidimensional scale), providing information that may have been overlooked had only one method been utilised. The purpose of evaluating the fit of a unidimensional model to each of the three subscales also enabled the assessment of whether each, or any, of the subscales could potentially be utilised as an independent scale. The use of a multidimensional model additionally enables the calibration of each subscale simultaneously, increasing measurement precision by including an assessment of the correlations between subscales. This advantage of multidimensional models is especially prominent when subscale length is limited or correlations between subscales are high [63], as is the case with RACES.

### Response category variability

A further assumption of polytomous Rasch models is that the data set to be analysed has acceptable response category variability to avoid unstable measures, inaccurate model fit indices, and incorrect inferences [64]. For measure stability it is helpful for the accuracy of model fit and for drawing inferences from the data [64]. This ensures the robustness of the estimates, or that similar estimates could be obtained with another sample from an equivalent population. A guideline for RSM is a minimum of 10 observations in each category accumulated across all relevant items (M. Linacre and R. Adams, personal communication, September 16, 2014). A smaller number of observations only at the item level can impact upon the capacity to accurately assess fit.

To assess the assumption of response category variability, we examined the number of responses in each category for each item. All data sets met the minimum criterion; however, the 21+ years data set had a total of seven items (i.e., 29 % of scale) without a response in each category and was therefore not considered to have sufficient response variability to enable accurate analysis, precluding Rasch analysis of this data set. The Primary School data set, the overall Community data set, and the 15–20 years data set were examined, thus strengthening our results by allowing exploration of the latent trait structure of the three subscales of RACES using Rasch analysis across age groups.

### Unidimensionality

A final underlying assumption of unidimensional Rasch models is that the data have a unidimensional structure [65]. The underlying multidimensionality of RACES [36] precluded examining the scale as a single unidimensional measure. Although multidimensional Rasch models exist, they are complex and limited software is available to facilitate flexible analysis [66, 67]. Hence, examination utilising a multidimensional model provided supplementary information, rather than acting as a central analysis. Each subscale was examined separately utilising the unidimensional RSM, as is appropriate when multiple subscales are assumed to tap a unidimensional construct [66].

Although CFA has disadvantages for evaluating underlying unidimensionality prior to undertaking Rasch analysis, it is common in psychological research [68]. Moreover, even when more advanced methods such as the TETRAD method, the Rasch model, or Parallel analysis are utilised to confirm unidimensionality, subjective judgment is required to determine underlying dimensionality [68]. CFA utilising a congeneric (one factor) measurement model was therefore considered sufficient to examine the underlying unidimensionality of each of the subscales prior to undertaking further Rasch analyses. Each subscale was assessed separately, with an evaluation of the fit of all items within each subscale performed.

## Results

### Unidimensionality

The unidimensionality of each subscale (AAS, RAS, and EAS) was examined utilising a separate congeneric (one factor) measurement model CFA for all data sets (Primary School, Community, and 15–20 years). The *χ*^2^ statistic indicated poor fit for a number of analyses. However, this statistic is sensitive to sample size and a number of alternative, less conservative, fit indices are available [69]. To avoid model misspecification multiple indices of fit were examined using widely accepted cut-off criteria [70]. CMIN/*df* is considered poor fit above 3.00 [71]; RMSEA poor fit above .10 [69] and good fit below .08 [72]; IFI good fit above .90 [73]; and SRMR good fit below .10 [74]. Each hypothesised factor for all data sets was considered to be of sufficient unidimensionality to undertake Rasch analysis (see Tables 3 and 4).

**Table 3:** RACES subscales CFA unidimensionality results

	Subscale	*χ* ^2^	*df*	*p*	CMIN/*df*	RMSEA	IFI	SRMR
Primary school	Accepting attitudes	177.65	65	<.001^***^	2.73^a^	.09^a^	0.86	.07^a^
	Racist attitudes	35.10	20	.020^*^	1.76^a^	.06^a^	0.96^a^	.05^a^
	Ethnocentric attitudes	1.25	2	.53^a^	0.63^a^	<.01^a^	1.01^a^	.02^a^
15–20 years	Accepting attitudes	137.82	65	<.001^***^	2.12^a^	.09^a^	0.92^a^	.05^a^
	Racist attitudes	40.79	20	.004^**^	2.04^a^	.08^a^	0.94^a^	.06^a^
	Ethnocentric attitudes	0.57	2	.75^a^	0.29^a^	<.01^a^	1.01^a^	<.01^a^
Community	Accepting attitudes	174.42	65	<.001^***^	2.68^a^	.08^a^	0.93^a^	.05^a^
	Racist attitudes	58.76	20	<.001^***^	2.94^a^	.09^a^	0.94^a^	.05^a^
	Ethnocentric attitudes	0.19	2	.91^a^	0.09^a^	<.01^a^	1.01^a^	<.01^a^

*Note:* ^*^ *p* < .05. ^**^ *p* < .01. ^***^ *p* < .001

^a^ denotes acceptable fit

**Table 4:** CFA congeneric (one factor) measurement model factor loadings for races subscales

Subscale	Item	Primary school	15–20 years	Community
AAS	I accept people from all backgrounds.	.74	.88	.81
	I have respect for people from all backgrounds.	.71	.81	.82
	People from all backgrounds are equal.	.67	.77	.71
	Having many different backgrounds in Australia is a good thing.	.64	.69	.66
	People from all backgrounds should be treated equally.	.64	.54	.63
	I live peacefully with people from all backgrounds.	.62	.68	.66
	I share with people from all backgrounds.	.62	.79	.77
	I like talking with people from all backgrounds.	.60	.74	.72
	I don’t tease people because of their background.	.49	.41	.46
	I stand up for people from all backgrounds.	.49	.48	.55
	We should be taught about all backgrounds in school.	.48	.55	.49
	I get upset if I hear racist comments about any background.	.47	.48	.48
	I don’t ignore people because of their background.	.43	.59	.66
RAS	People from some backgrounds are more violent than others.	.73	.76	.76
	I don’t trust people from some backgrounds.	.65	.86	.87
	People from some backgrounds are not friendly.	.65	.74	.74
	People from some backgrounds are more likely to get into trouble than others.	.60	.45	.44
	I don’t understand people from some backgrounds.	.55	.43	.50
	If people aren’t happy in Australia they should go back to their own country.	.53	.56	.62
	People from some backgrounds get more than they deserve.	.52	.22	.24
	If people don’t fit into Australian society they should change.	.43	.53	.51
EAS	I only feel comfortable around people from my background.	.73	.83	.78
	I only feel safe around people from my background.	.67	.76	.81
	Only people from my background understand me.	.59	.68	.66
	I only have friends from my background.	.50	.53	.55

*Note: AAS* accepting attitudes scale, *RAS* racist attitudes scale, *EAS* ethnocentric attitudes scale

### Unidimensional model fit

#### Primary school data set

All items on each subscale had acceptable Infit and Outfit. When Standardised values were examined EAS had acceptable fit, but AAS and RAS had several items of less than ideal fit. However, no items were removed due to the sensitivity of this index to sample size and the acceptable Infit and Outfit values across each item. Each of the reliability indices (separation reliability and EAP/PV reliability) indicated that all RACES subscales had acceptable reliability (i.e., > .70; [75]). EAP/PV reliability is the explained variance according to the estimated model divided by the total individuals variance [76]. As explained previously, Rasch models permit separation of the individual and item parameters. Separation reliability is a summary of ‘true’ separation as a ratio to separation including measurement error (the ratio of sample deviation, corrected for error, to the average estimation error), indicating how well a test can separate individuals by performance; it is comparable to the Kuder-Richardson Formula 20 measure of internal consistency [77].

#### 15–20 years data set

Several items across the subscales were of less than ideal fit when Standardised values were examined. However, one item (“I don’t tease people because of their background”) on AAS had unacceptable Infit and Outfit. Each of the reliability indices indicated that RAS and AAS had acceptable reliability. EAS had poor separation reliability, but acceptable EAP/PV reliability. The misfitting item from AAS was removed from further analysis with this data set following recommendations of initially removing underfitting items (i.e., > 1.5; [78]), and the RSM analysis was re-conducted.

All items on the subscale were of acceptable Infit and Outfit, although several fell outside the recommended Standardised values range. All items were retained, however, due to the sensitivity of the index, and the balance achieved with the current total RACES of 12 positive items and 12 negative items. This balance avoids response bias due to (1) the sensitivity of the attitudes under evaluation [79, 80] and (2) the tendency for participants to acquiesce, especially those with lower levels of general knowledge and cognitive sophistication (e.g., younger individuals and those with less formal education) [81]. It allows exploration of both positive (acceptance) and negative (racism) attitudes which are functionally independent (i.e., positive attitudes are stronger predictors of positive behaviours and negative attitudes are stronger predictors of negative behaviours) (cf. [19, 82]) and conceptually distinct [83].

#### Community data set

All items on EAS were of acceptable Infit and Outfit. AAS had one item (“I don’t tease people because of their background”) with undesirable Infit and Outfit and one item (“I get upset if I hear racist comments about any background”) with less than ideal Outfit. RAS had one item (“People from some backgrounds get more than they deserve”) with undesirable Infit. Several items across the subscales were of less than ideal fit when Standardised values were examined. However, due to the sensitivity of this index and acceptable Infit and Outfit values across most items, only one item (“I don’t tease people because of their background”) of poor fit across all indices was removed from further analysis with this data set, and the RSM analysis was re-conducted.

Two items had Outfit outside of the recommended range (“I get upset if I hear racist comments about any background” and “We should be taught about all backgrounds in school”). All other items were of acceptable Infit and Outfit. Several items were outside the recommended Standardised values range, but were retained due to (1) the Infit-Outfit discrepancies, with no items considered degrading of the measurement system (2) the sensitivity of the Standardised values index, and (3) the balance achieved with the current total RACES scale of 12 positive items and 12 negative items if no further items are removed.

#### Primary school data set re-analysis

Due to the potential value of a single scale containing precisely the same items to assess racism across age groups, the Primary School data set was re-assessed. One item problematic in both the 15–20 years and overall Community data sets (“I don’t tease people because of their background”), was removed from AAS and the RSM analysis was re-conducted. All items on the subscale were of acceptable Infit and Outfit. Although several items were outside the recommended Standardised values range, all items were retained due to reasons reported above.

The final model fit statistics for each data set and subscale are shown in Table 5 below.

**Table 5:** Unidimensional model fit indices for RACES subscales

Sub scale	Item	Infit			Standardised value			Outfit			Standardised value		
		PS	15–20	C	PS	15–20	C	PS	15–20	C	PS	15–20	C
AAS	I have respect for people from all backgrounds.	1.23	0.78	0.79	1.9	–1.7	−2.2	1.09	0.60	0.65	0.9	−4.0^a^	−4.6^a^
	I accept people from all backgrounds.	0.86	0.67	0.89	−1.4	−2.7^a^	−1.1	0.85	0.51	0.73	−1.5	−5.2^a^	−3.3^a^
	Having many different backgrounds in Australia is a good thing.	0.96	0.97	0.95	−0.3	−0.2	−0.5	0.93	0.95	0.88	−0.7	−0.4	−1.4
	People from all backgrounds should be treated equally.	0.74	1.16	1.00	−2.7^a^	1.2	0.0	0.72	1.10	0.93	−3.0^a^	0.8	−0.8
	I share with people from all backgrounds.	1.47	0.63	0.68	4.1^a^	−3.2^a^	−3.8^a^	1.33	0.69	0.89	2.8	−2.9^a^	−1.3
	People from all backgrounds are equal.	0.72	1.10	1.17	−3.0^a^	0.6	1.6	0.72	0.89	1.08	−2.8^a^	−1.0	0.9
	I live peacefully with people from all backgrounds.	0.92	0.96	0.91	−0.7	−0.2	−0.9	0.86	1.07	1.02	−1.4	0.6	0.3
	I like talking with people from all backgrounds.	1.14	0.89	0.86	1.4	−0.8	−1.4	1.18	0.85	0.82	1.8	−1.3	−2.2^a^
	We should be taught about all backgrounds in school.	0.77	1.36	1.42	−2.1^a^	2.5	3.8^a^	0.78	1.41	1.54^a^	−2.3^a^	3.2^a^	5.4
	I get upset if I hear racist comments about any background.	1.28	1.49	1.50	2.9	3.4^a^	4.8^a^	1.43	1.49	1.55^a^	3.8^a^	3.7^a^	5.4^a^
	I stand up for people from all backgrounds.	1.00	1.41	1.26	0.1	2.9	2.7	1.03	1.33	1.30	0.4	2.6	3.1^a^
	I don’t ignore people because of their background.	1.43	0.97	1.03	3.5^a^	−0.2	0.3	1.37	1.13	1.00	3.3^a^	0.9	0.0
RAS	People from some backgrounds are more violent than others.	0.79	0.85	0.92	−2.5^a^	−1.5	−1.1	0.79	0.85	0.91	−2.2^a^	−1.3	−1.1
	People from some backgrounds are not friendly.	0.81	0.85	0.83	−2.2^a^	−1.4	−2.3^a^	0.83	0.85	0.84	−1.8	−1.3	−1.9
	People from some backgrounds are more likely to get into trouble than others.	0.95	0.92	0.97	−0.6	−0.7	−0.3	0.96	0.93	0.98	−0.3	−0.5	−0.2
	I don’t trust people from some backgrounds.	0.87	0.82	0.76	−1.5	−1.7	−3.3^a^	0.87	0.80	0.75	−1.3	−1.8	−3.1^a^
	If people aren’t happy in Australia they should go back to their own country.	1.18	1.15	1.16	1.9	1.4	1.9	0.15	1.15	1.16	−1.4	1.3	1.7
	People from some backgrounds get more than they deserve.	1.16	1.48	1.51^a^	1.7	3.9^a^	5.4^a^	1.16	1.48	1.49	1.5	3.6^a^	4.9^a^
	I don’t understand people from some backgrounds.	1.01	0.93	0.95	0.1	−0.6	−0.7	0.99	0.95	0.95	−0.1	−0.4	−0.5
	If people don’t fit into Australian society they should change.	1.27	0.91	0.90	2.7	−0.8	−1.3	1.29	0.93	0.92	2.6	−0.6	−1.0
EAS	I only feel safe around people from my background.	0.93	0.91	0.83	−0.6	−0.7	−1.6	0.95	0.86	0.85	−0.5	−1.2	−1.7
	I only feel comfortable around people from my background.	0.88	0.85	1.01	−1.1	−1.2	0.1	0.87	1.03	0.88	−1.3	0.3	−1.4
	Only people from my background understand me.	1.10	1.11	1.10	0.9	0.8	0.9	1.09	1.03	1.06	0.9	0.3	0.7
	I only have friends from my background.	1.11	1.21	1.18	1.0	1.5	1.6	1.03	1.07	1.04	0.3	0.6	0.5

Note: *PS* primary school sample, *15-20* 15–20 years sample, *C* community sample, *AAS* accepting attitudes scale, *RAS* racist attitudes scale, *EAS* ethnocentric attitudes scale

^a^ denotes value outside of recommended range

### Unidimensional scale information

Rasch analysis enables graphical representations of item and total scale characteristics of the data. The Item Characteristic Curve (ICC) or Item Response Function (IRF) and the Expected Score Curve (ESC) are key graphical representations of the performance of items within a Rasch analysis. The Test Information Function (TIC) or Test Information Function (TIF) is a core graphical representation of the performance of the overall test or scale within a Rasch analysis. Due to space constraints, only the TIF for the Community data set subscales are depicted graphically in the main text of this article (additional figures displaying the alternate data set TIFs are presented in Additional file 1, available online). However, the performance of RACES overall scale and subscales are described in the context of each graphical representation below.

The ICC/IRF shows the probability of a correct response as a function of the trait level of an individual and provides a nuanced analysis of item categories. These graphs represent probability as a function of ability plotted along an S‐shaped curve, with low trait levels having a probability of close to zero and high trait levels having a probability of close to one. The leftmost ICCs are the items ‘easiest’ to endorse (i.e., individuals low to high on the latent trait would endorse) and the rightmost items are the most ‘difficult’ to endorse (i.e., only individuals high on the latent trait would endorse). For our purposes an ‘easy’ item would capture individuals with low to high levels of accepting attitudes, while a ‘difficult’ item would be endorsed only by individuals with high levels of attitudes of acceptance (or low levels of racist and ethnocentric attitudes).

Depending on the purpose of the test, it may be important to have most items with high (e.g., measures of psychopathology) or low (e.g., measures of intellectual impairment) ‘difficulty’ levels. Within any test or scale intended for an average population, items need to be of varying ‘difficulty’. These figures illustrate that each RACES subscale contains items ranging from ‘easy’ to endorse to ‘difficult’ to endorse. If utilised as an entire multidimensional scale, RACES contains items that provide information about and can discriminate between individuals from low to high on the latent trait. As RACES was designed for use with a normal (i.e., average) population (versus highly racist or highly accepting), the ICCs of each of the subscales would be appropriate if utilised in combination. Items from each of the subscales performed similarly across each of primary school children, adolescents, and adults.

The ESC shows the expected score given the trait level of an individual and enables an analysis of general fit. The leftmost ESCs are the ‘easiest’ items and the rightmost the most ‘difficult’ items. These figures illustrate that many of RACES items across each subscale performed as predicted by the underlying model. Importantly, items from the subscales performed similarly across each of primary school children, adolescents and adults.

The Item Information Curve (IIC) or Item Information Function (IIF) shows the range where an item is best at discriminating among individuals of a certain trait level. However, the TIC/TIF better represents the data as it provides an illustrative summary of the combined information for all items on each subscale. Like the IIC/IIF, the TIC/TIF shows the range where an overall test is best at discriminating among individuals of a certain trait level. Higher information denotes more precision (or reliability) for measuring a person’s trait level. The TIC/TIF for each Community data set subscale is shown in Fig. 1 below.

**Fig. 3:**
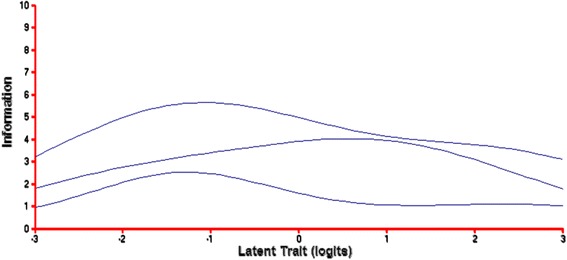
Community data set subscale TIFs. The upper most line represents the AAS, the middle line represents the RAS, and the lower most line represents the EAS. The TIF shows the range where each subscale provides the most information or at which trait level the subscale is best at discriminating among individuals. The left most latent trait represents individuals low on the latent trait and the right most latent trait represents individuals high on the latent trait

The upper most line represents AAS, the middle line RAS, and the lowest line EAS. As illustrated, each RACES subscale generally only contains items that provide information about, and is able to discriminate between, individuals either from low, moderate, or high on the latent trait. Nonetheless, if utilised as an entire multidimensional scale, RACES contains items that provide information enabling discrimination between individuals from low to high on the latent trait. As RACES was designed for use with a normal population, the TIC/TIFs of each of the subscales are appropriate when utilised in combination. Importantly, the subscales performed similarly across each of primary school children, adolescents and adults Fig. 3.

### Multidimensional model fit

The underlying structure of RACES as multi-scale was examined using multidimensional RSM analysis, to assess the between item multidimensionality of RACES with the aforementioned three subscale structure (i.e., 12-item AAS, 8-item RAS, and 4-item EAS). Data for the Primary School, Community, and 15–20 years data sets was collated in ACER ConQuest 3.0. Analysis using the RSM was undertaken for each data set for the overall scale with 24 items. Model fit was assessed utilising recommended criteria, as previously described. For each data set the *χ*^2^ statistic indicated a poor fit for the total RACES (Primary School: *χ*^2^ (21) = 314.79, *p* < .001; 15–20 years: *χ*^2^ (21) = 155.43, *p* < .001; Community: *χ*^2^ (21) = 323.94, *p* < .001). Moreover, several items across data sets were of less than ideal fit when Standardised values were examined. However, due to the sensitivity of these indices to sample size, other model fit indices were examined.

One item (“I don’t ignore people because of their background”) was of less than ideal Infit and Outfit for the Primary School data set. One item (“People from some backgrounds get more than they deserve”) had undesirable Infit and Outfit for the 15–20 years data set; two further items (“I get upset if I hear racist comments about any background” and “I accept people from all backgrounds”) had less than ideal Outfit. For the Community date set, one item (“People from some backgrounds get more than they deserve”) had undesirable Infit and Outfit, and one further item (“I get upset if I hear racist comments about any background”) had less than ideal Outfit. All other items across the data sets were of acceptable Infit and Outfit and no items were considered to degrade the measurement system (as per [53]). The multidimensional model fit statistics are displayed in Table 6 below.

**Table 6:** Multidimensional model fit indices for RACES subscales

Sub scale	Item	Infit			Standardised value			Outfit			Standardised value		
		PS	15–20	C	PS	15–20	C	PS	15–20	C	PS	15–20	C
AAS	I have respect for people from all backgrounds.	0.91	0.76	0.73	−0.8	−1.9	−3.0^a^	0.83	0.63	0.61	−1.7	−3.7^a^	−5.2^a^
	I accept people from all backgrounds.	0.69	0.67	0.82	−3.1^a^	−2.7^a^	−1.8	0.70	0.49^a^	0.67	−3.3^a^	−5.4^a^	−4.3^a^
	Having many different backgrounds in Australia is a good thing.	0.86	0.86	0.82	−1.5	−1.0	−1.9	0.88	0.88	0.79	−1.2	−1.1	−2.5^a^
	People from all backgrounds should be treated equally.	0.91	1.16	0.94	−0.8	1.1	−0.6	0.85	1.46	0.94	−1.5	3.5^a^	−0.7
	I share with people from all backgrounds.	0.74	0.62	0.60	−2.8^a^	−3.4^a^	−5.0^a^	0.72	0.63	0.73	−3.0^a^	−3.6^a^	−3.4^a^
	People from all backgrounds are equal.	0.96	1.02	1.09	−0.3	0.2	0.9	0.96	0.86	1.08	−0.3	−1.2	1.0
	I live peacefully with people from all backgrounds.	0.99	0.88	0.83	0.0	−0.9	−1.9	0.99	1.02	0.91	−0.1	0.2	−1.1
	I like talking with people from all backgrounds.	0.75	0.83	0.74	−2.6^a^	−1.4	−2.9^a^	0.76	0.80	0.72	−2.5^a^	−1.8	−3.5^a^
	We should be taught about all backgrounds in school.	1.29	1.26	1.32	2.7	1.9	3.0	1.32	1.32	1.43	2.9	2.5	4.4^a^
	I get upset if I hear racist comments about any background.	1.12	1.50	1.44	1.2	3.5^a^	4.3^a^	1.15	1.55^a^	1.59^a^	1.4	4.1^a^	5.8^a^
	I stand up for people from all backgrounds.	1.00	1.29	1.13	0.1	2.1	1.5	1.11	1.28	1.13	1.1	2.2	1.5
	I don’t ignore people because of their background.	1.79^a^	1.07	0.91	6.2^a^	0.5	−0.9	1.82^a^	1.05	0.92	6.6^a^	0.5	−0.9
RAS	People from some backgrounds are more violent than others.	0.80	0.99	0.99	−2.3^a^	0.0	0.0	0.83	1.03	1.01	−1.7	0.3	0.1
	People from some backgrounds are not friendly.	0.82	0.90	0.90	−2.1^a^	−0.9	−1.1	0.84	0.89	0.90	−1.6	−0.9	−1.2
	People from some backgrounds are more likely to get into trouble than others.	1.01	1.09	1.08	0.1	0.8	1.0	1.03	1.13	1.13	0.3	1.1	1.4
	I don’t trust people from some backgrounds.	0.89	0.95	0.84	−1.2	−0.4	−2.0	0.89	0.93	0.82	−1.1	−0.6	−2.1^a^
	If people aren’t happy in Australia they should go back to their own country.	1.23	1.23	1.27	2.3	1.9	3.0	1.19	1.21	1.24	1.8	1.7	2.6
	People from some backgrounds get more than they deserve.	1.22	1.60^a^	1.69^a^	2.2	4.4^a^	7.0^a^	1.20	1.58^a^	1.72^a^	1.9	4.6^a^	6.8^a^
	I don’t understand people from some backgrounds.	1.05	0.98	1.01	0.5	−0.1	0.1	1.04	1.02	1.03	0.5	0.2	0.4
	If people don’t fit into Australian society they should change.	1.34	1.00	0.99	3.2^a^	0.0	−0.1	1.37	0.98	1.00	3.3^a^	−0.1	0.0
EAS	I only feel safe around people from my background.	0.87	0.72	0.67	−1.3	−2.6^a^	−3.8^a^	0.89	0.74	0.69	−1.1	−2.2^a^	−4.0^a^
	I only feel comfortable around people from my background.	0.88	0.80	0.97	−1.2	−1.7	−0.3	0.86	0.78	0.99	−1.4	−2.0	−0.1
	Only people from my background understand me.	1.15	1.03	0.95	1.4	0.2	−0.5	1.14	0.98	0.98	1.4	−0.1	−0.2
	I only have friends from my background.	1.08	1.03	1.05	0.7	0.3	0.5	1.04	1.01	1.09	0.5	0.2	1.0

Note: *PS* primary school sample, *15–20* 15–20 years sample, *C* community sample, *AAS* accepting attitudes scale, *RAS* racist attitudes scale, *EAS* ethnocentric attitudes scale

^a^ denotes value outside of recommended range

### Multidimensional scale information

Graphical representations of the data illustrate item and combined total scale characteristics (additional figures displaying the Community data set data are presented in Additional file 1, which is available online; all other figures are available upon request from the lead author). The ICCs illustrate that the multidimensional RACES contains items that range from ‘easy’ to endorse to ‘difficult’ to endorse. These items performed similarly across each of primary school children, adolescents, and adults. The ESCs illustrate that many of RACES items performed as predicted by the underlying multidimensional model. These items performed similarly for each of primary school children, adolescents, and adults.

## Discussion

The aim of the project reported in this article was to refine and validate an attitudinal measure of racial, ethnic, cultural, and religious acceptance, for use as a proxy to quantify racist attitudes (see [36]). The end goal was to produce an instrument for use in community-wide anti-racism and pro-diversity initiatives, to assist in evaluating, refining, and improving their effectiveness, so to contribute to programs to reduce racism and increase acceptance of difference throughout Australia. It was hoped that in turn inequities in health outcomes across Australia’s diverse racial, ethnic, cultural, and religious groups could be redressed.

Insufficient attempts to reduce racism can lead to an intensification of racist attitudes [84, 85]. Because of this, it is crucial for racism reduction interventions to be based on a sound theoretical framework, as demonstrated over decades of research [9, 84, 86–89]. Yet a recent review of 50 years of diversity training demonstrated that in most cases programs are considered effective contingent upon the number of people trained, not by accurately evaluating their efficacy [90]. Without appropriate evaluation and demonstration of the efficacy of such interventions, anti-racism and pro-diversity programs cannot be widely disseminated and are therefore neither meaningful nor useful to the community at large.

A principal concern in developing and validating RACES was the lack of confidence in the capability of existing instruments to capture the varied forms of racism experienced by individuals of diverse groups in Australia. This is essential, as distinct groups often report diverse aspects and dissimilar experiences of racism and discrimination [91]. By adopting a comprehensive process to develop and validate RACES, the measure can be used with multiple groups across the lifespan.

The present research demonstrated the robust reliability and validity of RACES, confirming the utility of the measure. Overall, RACES has a number of key advantages as a measure of racist attitudes in Australia. RACES was developed for, and validated in, the contemporary Australian social context, with previous development phases ensuring that the items were based on real experiences, understandings, and conceptualisations, utilising a mixed-methods approach. This contrasts with many measures that draw on secondary data or uncritically re-word or adapt existing scales and rely solely upon quantitative methods. Unlike any existing measure of racist attitudes, RACES was assessed and refined utilising both CTT and IRT, giving greater confidence in its factorial validity. The Rasch analyses support the overall factorial and construct validity of the 24-item RACES across primary school children, adolescents, and adults, and indicate that RACES is a reliable three-dimensional scale of Accepting Attitudes (12 items), Racist Attitudes (8 items), and Ethnocentric Attitudes (4 items). RACES also provides information about, and discriminates between, individuals across the range of the latent traits of racism, acceptance, and cultural-ethnocentrism. Finally, in contrast to previous measures of racism in Australia, RACES was designed for assessing attitudes towards all racial, ethnic, cultural, and religious groups and has been shown to be reliable and valid across children, adolescents, and adults.

### Limitations

Although participants were sought from around Australia and across the range of adolescent and adult ages for the Community data set, the sample was predominantly from Victoria and the average age was quite young, limiting the generalisability of the results. Minimum sample sizes for factor analysis and other analyses were met, but replication and additional data from larger samples would enhance confidence in the results. Invalid responses may also have biased the results, although inspection of removed cases revealed that most missing data was from latter parts of the survey, suggesting that technical difficulties led to participant non-completion, rather than being characteristic of the participants. Some scale characteristics were less than ideal (e.g., fit indices) and therefore require confirmation with alternate populations. We did not remove items based on stringent cut-points due to the limited sample available, but there is the potential that findings are an artefact of the participants, reinforcing the need for replication. Finally, strong consistency was found across age groups, but results were based on an unbalanced overall scale (i.e., 12, 8, and 4 items), which may bias findings utilising the total scale score. Moreover, the failure of the 21+ years data set to meet minimum requirements for independent analysis casts some doubt on the uniformity found across age groups and hence requires further exploration. The brief length of the EAS also raises some concern due to the potential for short tests to lead to less accurate estimation in Rasch models [92, 93], although alternative research has demonstrated the accuracy of Rasch estimation for tests as short as five items [92, 94].

### Implications for practice

Prior to its wide dissemination to evaluate anti-racism and pro-diversity initiatives, future research is needed to confirm the psychometric properties of the new measure in alternate contexts and populations. Regardless, there are significant advantages of RACES over existing tools. RACES can be used to: a) evaluate the relationship between racism and other variables, b) track changes in racist attitudes over time, c) compare racist attitudes across groups, and d) evaluate the effect of anti-racism or pro-diversity initiatives. If the robust validity of the measure is confirmed in prospective research, potential gender, SES, and other demographic differences might be explored, so enhancing our understanding of racism in Australia. The most important use of RACES is its potential to assess the effectiveness of racism-reduction programs, by evaluating the attitudes of participants prior to and after intervention. Such evaluation would provide a strong evidence base for initiatives to be developed, refined, and extended to reduce community levels of racism. Due to its development stages predominantly involving youth, RACES has particular potential for effective use with school- or other youth-based initiatives.

## Conclusion

Racism is a significant challenge in contemporary Australian society due to the potential and significant negative impact on a range of health, social, psychological, and economic outcomes of the diverse racial, ethnic, cultural, and religious groups within Australia. Various interventions have attempted to reduce racism, increase acceptance of diversity, and address health inequities. However, confident conclusions about the effectiveness of such initiatives have not been able to be drawn, because of the absence of validated and standardised measures of racism appropriate for the diverse Australian population. The present project aspired to redress this issue and answer the appeals of previous researchers by working to inform developmentally targeted racism-reduction programs. RACES was designed to evaluate such initiatives and early validity findings offer solid foundations for, and confidence in, the instrument. Although follow up work is needed, RACES can be employed in a meaningful and useful manner to assist with the evaluation, and consequent targeted improvement, of innovative intervention programs for populations across the lifespan. Such appraisals would provide a strong evidence base for initiatives to reduce community levels of racism and in turn inequities in health outcomes across all racial, ethnic, cultural, and religious groups within Australia.

## Additional file

Additional file 1: The Australian Racism, Acceptance, and Cultural-Ethnocentrism Scale Appendix. Appendix of additional figures not included in main text. (DOCX 906 kb)

## Footnotes

1 Internal consistency reliability demonstrates that each item relates to each other item in the scale.

2 Factorial validity demonstrates that the identified factor structure is valid in respect to the underlying theoretical model.

3 Construct validity is an overall measure of validity that encompasses all other forms of reliability and validity. Construct validity demonstrates that the instrument measures what it purports to measure. In other words, the measure performs as it is expected to perform based on the overarching theory upon which it is based.

4 Convergent validity demonstrates that the measure is related to concepts it would be expected to be related to, or alternatively that results from two groups which would be expected to have similar results are related.

5 Discriminant validity demonstrates that the measure is unrelated to concepts it would be expected to be unrelated to, or alternatively that results from two groups which would be expected to have different results are different.
