# Supplementary material for: The Australian Racism, Acceptance, and Cultural-Ethnocentrism Scale (RACES): item response theory findings
Source: Int J Equity Health. 2016 Mar 17;15:49. doi: 10.1186/s12939-016-0338-4 (PMC4794855; doi:10.1186/s12939-016-0338-4)
Supplement: Additional file 1: — The Australian Racism, Acceptance, and Cultural-Ethnocentrism Scale Appendix. Appendix of additional figures not included in main text. (DOCX 906 kb) [file 12939_2016_338_MOESM1_ESM.docx]

**Appendix**


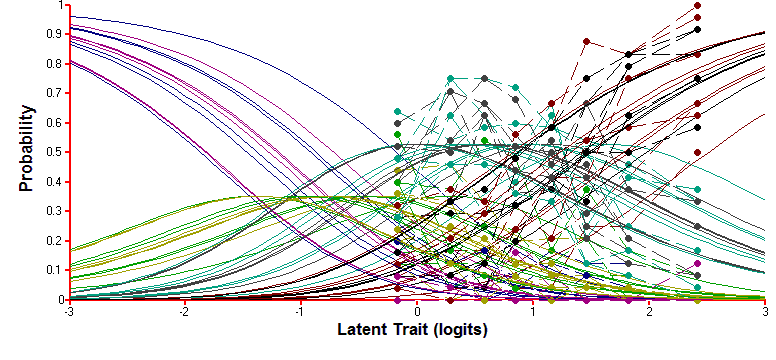


*Figure A1.* Primary school data set Accepting Attitudes Scale ICCs. The x-axis indicates the probability of a response. The y-axis indicates the level of the latent trait. The left most items will be endorsed by individuals low to high on the latent trait. The right most items will only be endorsed by individuals high on the latent trait. Each line represents one item. The smooth lines represent the ICCs predicted by the model. The broken lines represent the actual data. The cluster of purple lines represent the response option “Strongly Disagree”; the cluster of green lines represent the response option “Disagree”; the cluster of blue lines represent the response option “Agree”; the cluster of red lines represent the response option “Strongly Agree”.


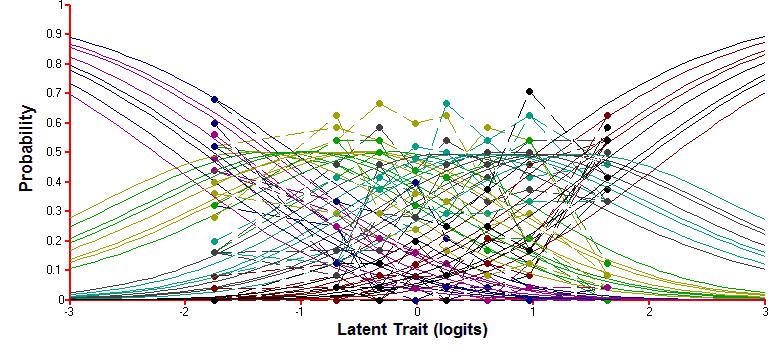


*Figure A2.* Primary school data set Racist Attitudes Scale ICCs. The x-axis indicates the probability of a response. The y-axis indicates the level of the latent trait. The left most items will be endorsed by individuals low to high on the latent trait. The right most items will only be endorsed by individuals high on the latent trait. Each line represents one item. The smooth lines represent the ICCs predicted by the model. The broken lines represent the actual data. The cluster of purple lines represent the response option “Strongly Disagree”; the cluster of green lines represent the response option “Disagree”; the cluster of blue lines represent the response option “Agree”; the cluster of red lines represent the response option “Strongly Agree”.


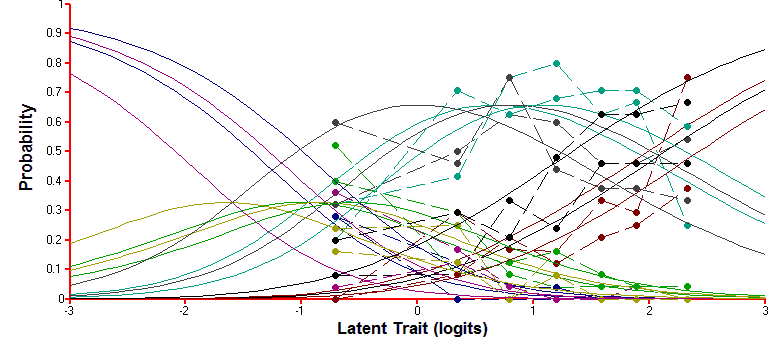


*Figure A3.* Primary school data set Ethnocentric Attitudes Scale ICCs. The x-axis indicates the probability of a response. The y-axis indicates the level of the latent trait. The left most items will be endorsed by individuals low to high on the latent trait. The right most items will only be endorsed by individuals high on the latent trait. Each line represents one item. The smooth lines represent the ICCs predicted by the model. The broken lines represent the actual data. The cluster of purple lines represent the response option “Strongly Disagree”; the cluster of green lines represent the response option “Disagree”; the cluster of blue lines represent the response option “Agree”; the cluster of red lines represent the response option “Strongly Agree”.


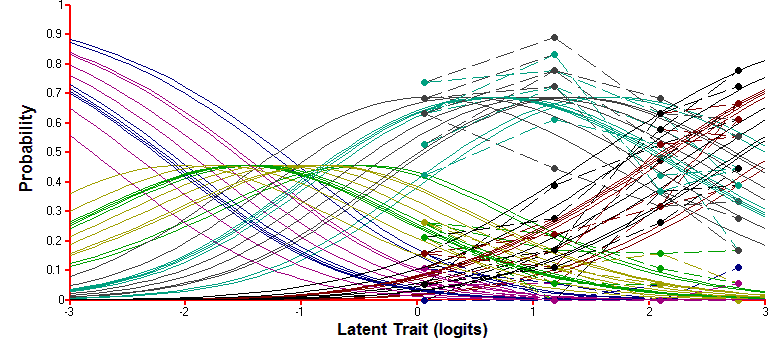


*Figure A4.* 15-20 years data set Accepting Attitudes Scale ICCs. The x-axis indicates the probability of a response. The y-axis indicates the level of the latent trait. The left most items will be endorsed by individuals low to high on the latent trait. The right most items will only be endorsed by individuals high on the latent trait. Each line represents one item. The smooth lines represent the ICCs predicted by the model. The broken lines represent the actual data. The cluster of purple lines represent the response option “Strongly Disagree”; the cluster of green lines represent the response option “Disagree”; the cluster of blue lines represent the response option “Agree”; the cluster of red lines represent the response option “Strongly Agree”.


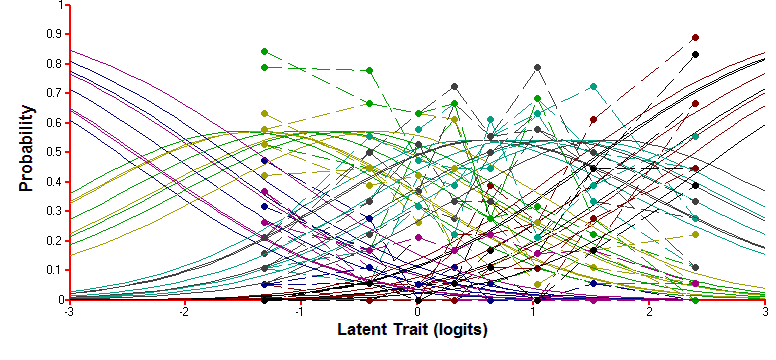


*Figure A5.* 15-20 years data set Racist Attitudes Scale ICCs. The x-axis indicates the probability of a response. The y-axis indicates the level of the latent trait. The left most items will be endorsed by individuals low to high on the latent trait. The right most items will only be endorsed by individuals high on the latent trait. Each line represents one item. The smooth lines represent the ICCs predicted by the model. The broken lines represent the actual data. The cluster of purple lines represent the response option “Strongly Disagree”; the cluster of green lines represent the response option “Disagree”; the cluster of blue lines represent the response option “Agree”; the cluster of red lines represent the response option “Strongly Agree”.


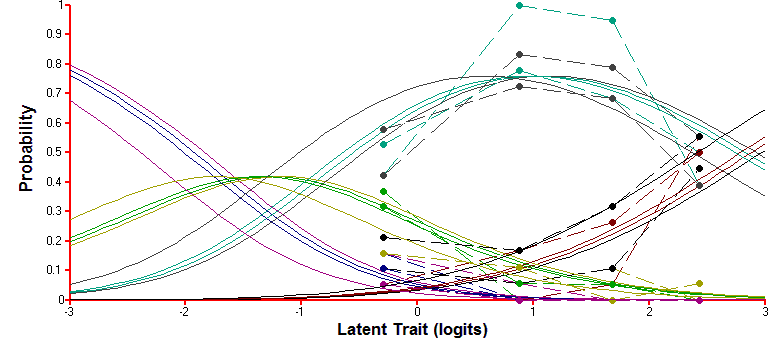


*Figure A6.* 15-20 years data set Ethnocentric Attitudes Scale ICCs. The x-axis indicates the probability of a response. The y-axis indicates the level of the latent trait. The left most items will be endorsed by individuals low to high on the latent trait. The right most items will only be endorsed by individuals high on the latent trait. Each line represents one item. The smooth lines represent the ICCs predicted by the model. The broken lines represent the actual data. The cluster of purple lines represent the response option “Strongly Disagree”; the cluster of green lines represent the response option “Disagree”; the cluster of blue lines represent the response option “Agree”; the cluster of red lines represent the response option “Strongly Agree”.


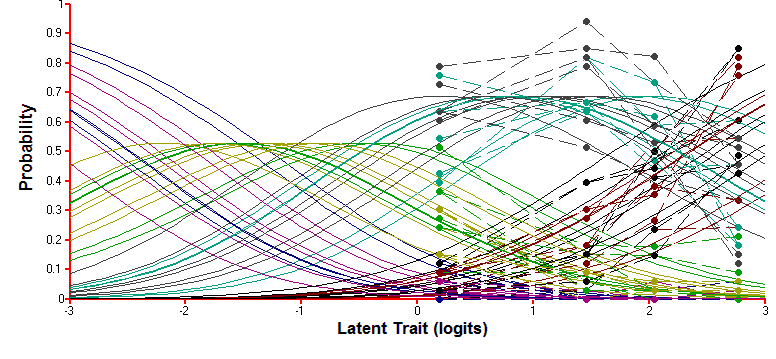


*Figure A7.* Community data set Accepting Attitudes Scale ICCs. The x-axis indicates the probability of a response. The y-axis indicates the level of the latent trait. The left most items will be endorsed by individuals low to high on the latent trait. The right most items will only be endorsed by individuals high on the latent trait. Each line represents one item. The smooth lines represent the ICCs predicted by the model. The broken lines represent the actual data. The cluster of purple lines represent the response option “Strongly Disagree”; the cluster of green lines represent the response option “Disagree”; the cluster of blue lines represent the response option “Agree”; the cluster of red lines represent the response option “Strongly Agree”.


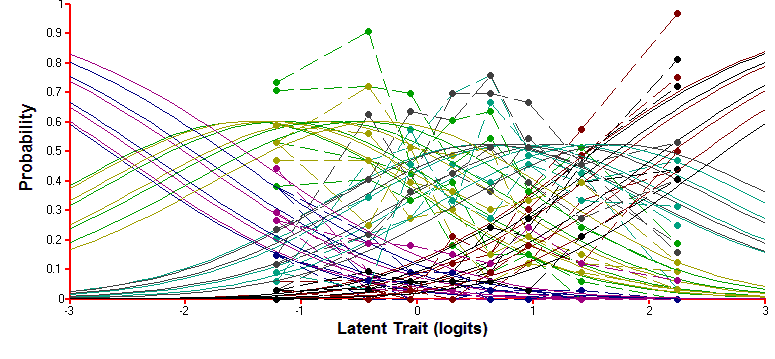


*Figure A8.* Community data set Racist Attitudes Scale ICCs. The x-axis indicates the probability of a response. The y-axis indicates the level of the latent trait. The left most items will be endorsed by individuals low to high on the latent trait. The right most items will only be endorsed by individuals high on the latent trait. Each line represents one item. The smooth lines represent the ICCs predicted by the model. The broken lines represent the actual data. The cluster of purple lines represent the response option “Strongly Disagree”; the cluster of green lines represent the response option “Disagree”; the cluster of blue lines represent the response option “Agree”; the cluster of red lines represent the response option “Strongly Agree”.


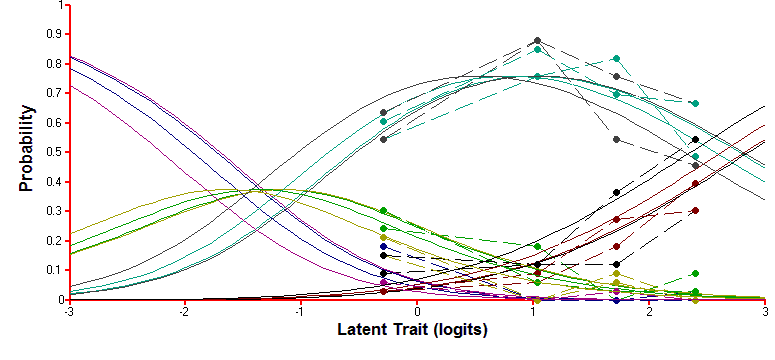


*Figure A9.* Community data set Ethnocentric Attitudes Scale ICCs. The x-axis indicates the probability of a response. The y-axis indicates the level of the latent trait. The left most items will be endorsed by individuals low to high on the latent trait. The right most items will only be endorsed by individuals high on the latent trait. Each line represents one item. The smooth lines represent the ICCs predicted by the model. The broken lines represent the actual data. The cluster of purple lines represent the response option “Strongly Disagree”; the cluster of green lines represent the response option “Disagree”; the cluster of blue lines represent the response option “Agree”; the cluster of red lines represent the response option “Strongly Agree”.


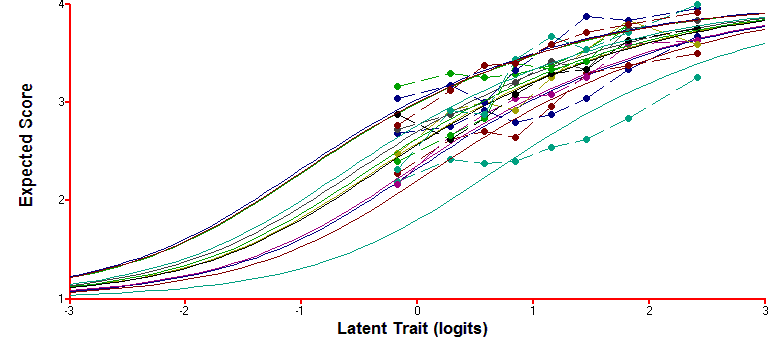


*Figure A10.* Primary school data set Accepting Attitudes Scale ESCs. The x-axis indicates the expected response (i.e., 1 = “Strongly Disagree”, 2 = “Disagree”, 3 = “Agree”, and 4 = “Strongly Agree”) predicted by the model. The y-axis indicates the level of the latent trait. Each individual line represents the ESC of one item. The smooth lines represent the expected score, given the trait level of an individual. The broken lines represent the actual data. The left most ESCs are the items that will be endorsed by individuals low to high on the latent trait. The right most ESCs are the items that will only be endorsed by individuals high on the latent trait.


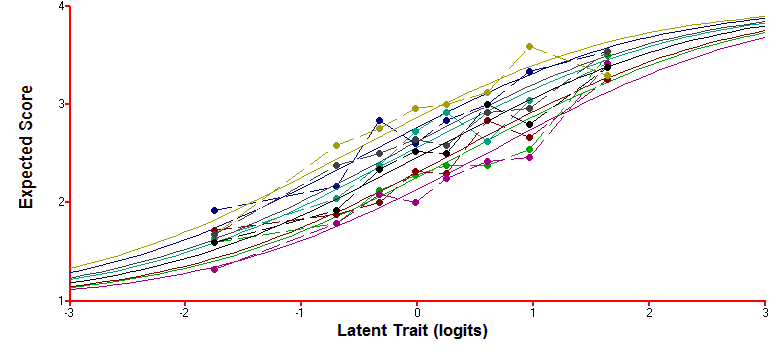


*Figure A11.* Primary school data set Racist Attitudes Scale ESCs. The x-axis indicates the expected response (i.e., 1 = “Strongly Disagree”, 2 = “Disagree”, 3 = “Agree”, and 4 = “Strongly Agree”) predicted by the model. The y-axis indicates the level of the latent trait. Each individual line represents the ESC of one item. The smooth lines represent the expected score, given the trait level of an individual. The broken lines represent the actual data. The left most ESCs are the items that will be endorsed by individuals low to high on the latent trait. The right most ESCs are the items that will only be endorsed by individuals high on the latent trait.


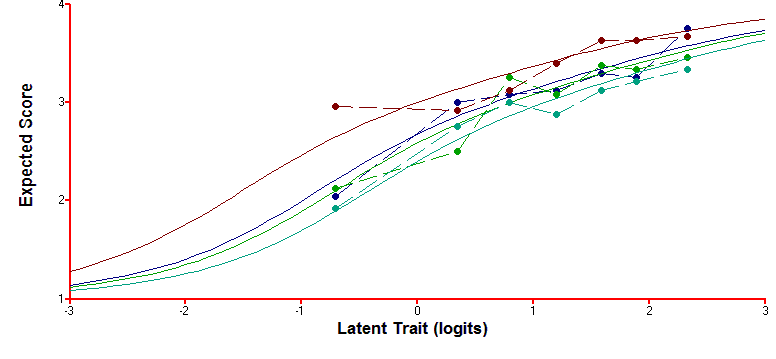


*Figure A12.* Primary school data set Ethnocentric Attitudes Scale ESCs. The x-axis indicates the expected response (i.e., 1 = “Strongly Disagree”, 2 = “Disagree”, 3 = “Agree”, and 4 = “Strongly Agree”) predicted by the model. The y-axis indicates the level of the latent trait. Each individual line represents the ESC of one item. The smooth lines represent the expected score, given the trait level of an individual. The broken lines represent the actual data. The left most ESCs are the items that will be endorsed by individuals low to high on the latent trait. The right most ESCs are the items that will only be endorsed by individuals high on the latent trait.


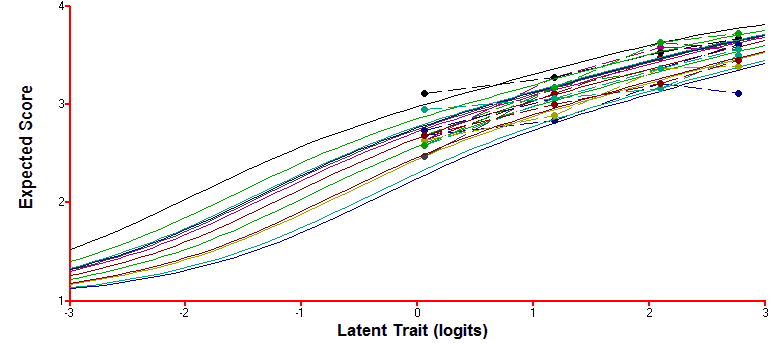


*Figure A13.* 15-20 years data set Accepting Attitudes Scale ESCs. The x-axis indicates the expected response (i.e., 1 = “Strongly Disagree”, 2 = “Disagree”, 3 = “Agree”, and 4 = “Strongly Agree”) predicted by the model. The y-axis indicates the level of the latent trait. Each individual line represents the ESC of one item. The smooth lines represent the expected score, given the trait level of an individual. The broken lines represent the actual data. The left most ESCs are the items that will be endorsed by individuals low to high on the latent trait. The right most ESCs are the items that will only be endorsed by individuals high on the latent trait.


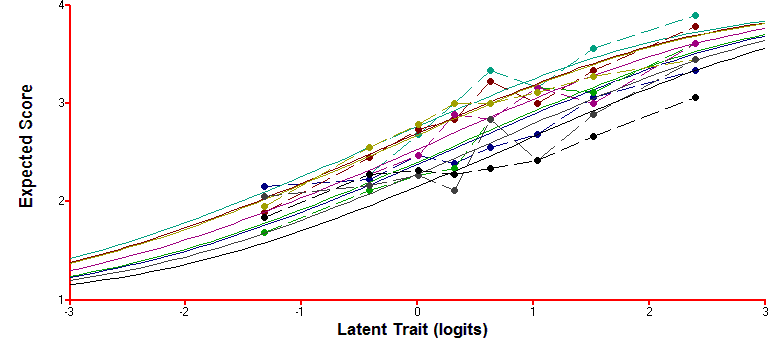


*Figure A14.* 15-20 years data set Racist Attitudes Scale ESCs. The x-axis indicates the expected response (i.e., 1 = “Strongly Disagree”, 2 = “Disagree”, 3 = “Agree”, and 4 = “Strongly Agree”) predicted by the model. The y-axis indicates the level of the latent trait. Each individual line represents the ESC of one item. The smooth lines represent the expected score, given the trait level of an individual. The broken lines represent the actual data. The left most ESCs are the items that will be endorsed by individuals low to high on the latent trait. The right most ESCs are the items that will only be endorsed by individuals high on the latent trait.


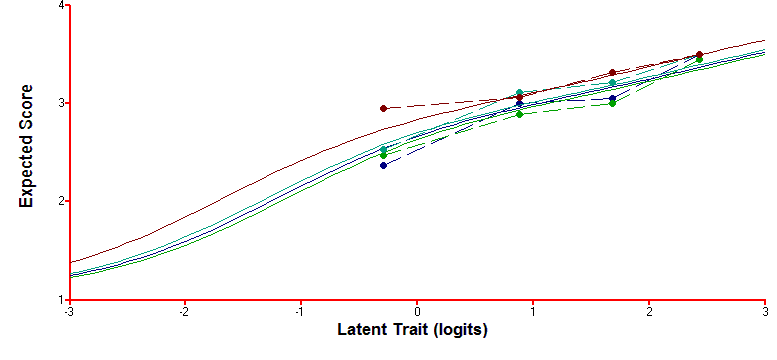


*Figure A15.* 15-20 years data set Ethnocentric Attitudes Scale ESCs. The x-axis indicates the expected response (i.e., 1 = “Strongly Disagree”, 2 = “Disagree”, 3 = “Agree”, and 4 = “Strongly Agree”) predicted by the model. The y-axis indicates the level of the latent trait. Each individual line represents the ESC of one item. The smooth lines represent the expected score, given the trait level of an individual. The broken lines represent the actual data. The left most ESCs are the items that will be endorsed by individuals low to high on the latent trait. The right most ESCs are the items that will only be endorsed by individuals high on the latent trait.


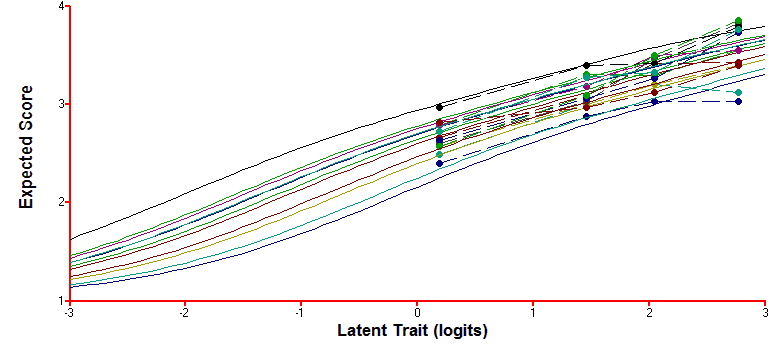


*Figure A16.* Community data set Accepting Attitudes Scale ESCs. The x-axis indicates the expected response (i.e., 1 = “Strongly Disagree”, 2 = “Disagree”, 3 = “Agree”, and 4 = “Strongly Agree”) predicted by the model. The y-axis indicates the level of the latent trait. Each individual line represents the ESC of one item. The smooth lines represent the expected score, given the trait level of an individual. The broken lines represent the actual data. The left most ESCs are the items that will be endorsed by individuals low to high on the latent trait. The right most ESCs are the items that will only be endorsed by individuals high on the latent trait.


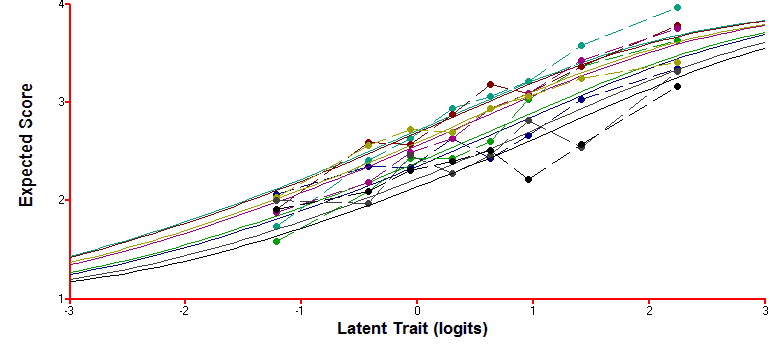


*Figure A17.* Community data set Racist Attitudes Scale ESCs. The x-axis indicates the expected response (i.e., 1 = “Strongly Disagree”, 2 = “Disagree”, 3 = “Agree”, and 4 = “Strongly Agree”) predicted by the model. The y-axis indicates the level of the latent trait. Each individual line represents the ESC of one item. The smooth lines represent the expected score, given the trait level of an individual. The broken lines represent the actual data. The left most ESCs are the items that will be endorsed by individuals low to high on the latent trait. The right most ESCs are the items that will only be endorsed by individuals high on the latent trait.


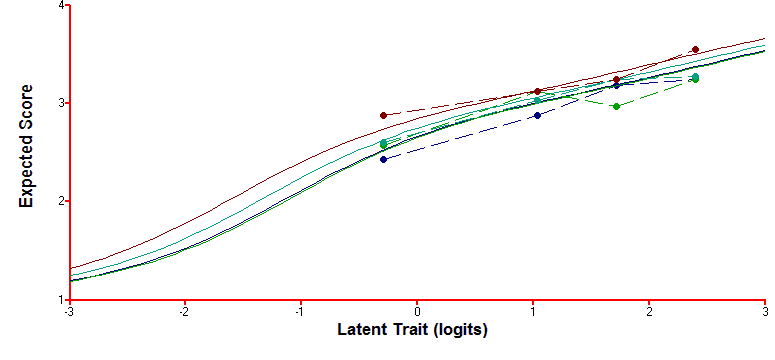


*Figure A18.* Community data set Ethnocentric Attitudes Scale ESCs. The x-axis indicates the expected response (i.e., 1 = “Strongly Disagree”, 2 = “Disagree”, 3 = “Agree”, and 4 = “Strongly Agree”) predicted by the model. The y-axis indicates the level of the latent trait. Each individual line represents the ESC of one item. The smooth lines represent the expected score, given the trait level of an individual. The broken lines represent the actual data. The left most ESCs are the items that will be endorsed by individuals low to high on the latent trait. The right most ESCs are the items that will only be endorsed by individuals high on the latent trait.

*Figure A19.* Primary school data set subscale TIFs. The upper most line represents AAS, the middle line represents RAS, and the lower most line represents EAS. The TIF shows the range where each subscale provides the most information or at which trait level the subscale is best at discriminating among individuals. The left most latent trait represents individuals low on the latent trait and the right most latent trait represents individuals high on the latent trait.

*Figure A20.* 15-20 years data set subscale TIFs. The upper most line represents AAS, the middle line represents RAS, and the lower most line represents EAS. The TIF shows the range where each subscale provides the most information or at which trait level the subscale is best at discriminating among individuals. The left most latent trait represents individuals low on the latent trait and the right most latent trait represents individuals high on the latent trait.

*Figure A21.* Community data set subscale TIFs. The upper most line represents AAS, the middle line represents RAS, and the lower most line represents EAS. The TIF shows the range where each subscale provides the most information or at which trait level the subscale is best at discriminating among individuals. The left most latent trait represents individuals low on the latent trait and the right most latent trait represents individuals high on the latent trait.


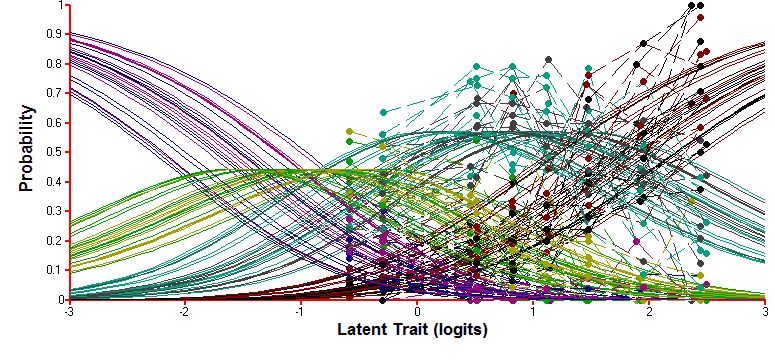


*Figure A22.* Primary school data set multidimensional RACES ICCs. The x-axis indicates the probability of a response. The y-axis indicates the level of the latent trait. The left most items will be endorsed by individuals low to high on the latent trait. The right most items will only be endorsed by individuals high on the latent trait. Each line represents one item. The smooth lines represent the ICCs predicted by the model. The broken lines represent the actual data. The cluster of purple lines represent the response option “Strongly Disagree”; the cluster of green lines represent the response option “Disagree”; the cluster of blue lines represent the response option “Agree”; the cluster of red lines represent the response option “Strongly Agree”.


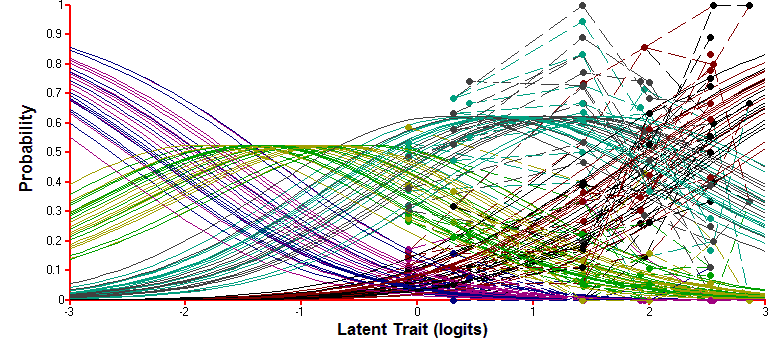


*Figure A23.* 15-20 years data set multidimensional RACES ICCs. The x-axis indicates the probability of a response. The y-axis indicates the level of the latent trait. The left most items will be endorsed by individuals low to high on the latent trait. The right most items will only be endorsed by individuals high on the latent trait. Each line represents one item. The smooth lines represent the ICCs predicted by the model. The broken lines represent the actual data. The cluster of purple lines represent the response option “Strongly Disagree”; the cluster of green lines represent the response option “Disagree”; the cluster of blue lines represent the response option “Agree”; the cluster of red lines represent the response option “Strongly Agree”.


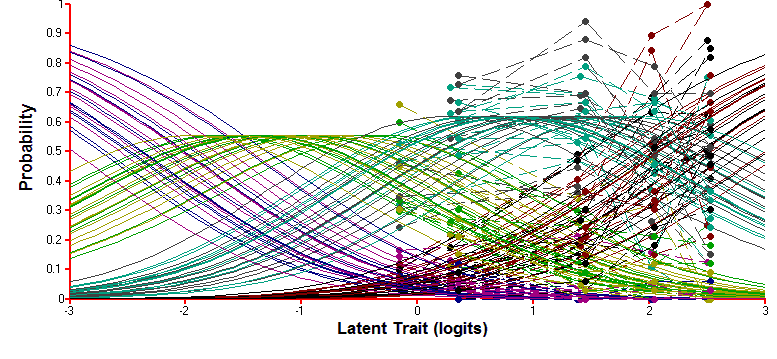


*Figure A24.* Community data set multidimensional RACES ICCs. The x-axis indicates the probability of a response. The y-axis indicates the level of the latent trait. The left most items will be endorsed by individuals low to high on the latent trait. The right most items will only be endorsed by individuals high on the latent trait. Each line represents one item. The smooth lines represent the ICCs predicted by the model. The broken lines represent the actual data. The cluster of purple lines represent the response option “Strongly Disagree”; the cluster of green lines represent the response option “Disagree”; the cluster of blue lines represent the response option “Agree”; the cluster of red lines represent the response option “Strongly Agree”.


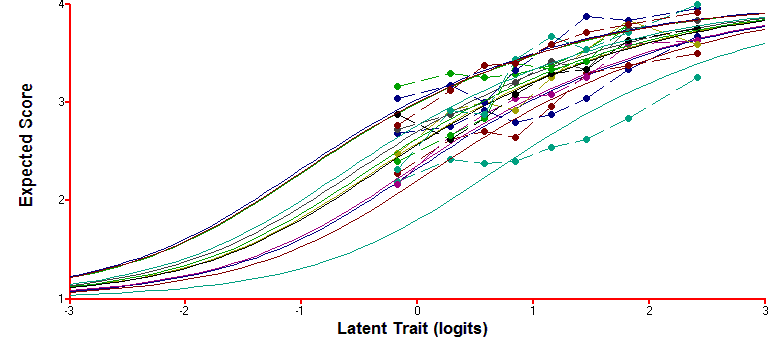


*Figure A25.* Primary school data set multidimensional RACES ESCs. The x-axis indicates the expected response (i.e., 1 = “Strongly Disagree”, 2 = “Disagree”, 3 = “Agree”, and 4 = “Strongly Agree”) predicted by the model. The y-axis indicates the level of the latent trait. Each individual line represents the ESC of one item. The smooth lines represent the expected score, given the trait level of an individual. The broken lines represent the actual data. The left most ESCs are the items that will be endorsed by individuals low to high on the latent trait. The right most ESCs are the items that will only be endorsed by individuals high on the latent trait.


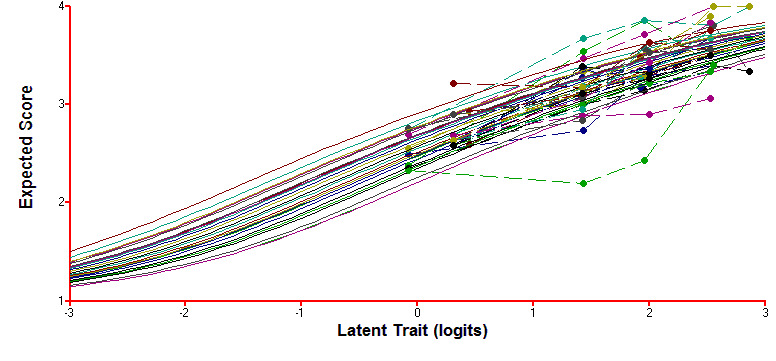


*Figure A26.* 15-20 years data set multidimensional RACES ESCs. The x-axis indicates the expected response (i.e., 1 = “Strongly Disagree”, 2 = “Disagree”, 3 = “Agree”, and 4 = “Strongly Agree”) predicted by the model. The y-axis indicates the level of the latent trait. Each individual line represents the ESC of one item. The smooth lines represent the expected score, given the trait level of an individual. The broken lines represent the actual data. The left most ESCs are the items that will be endorsed by individuals low to high on the latent trait. The right most ESCs are the items that will only be endorsed by individuals high on the latent trait.


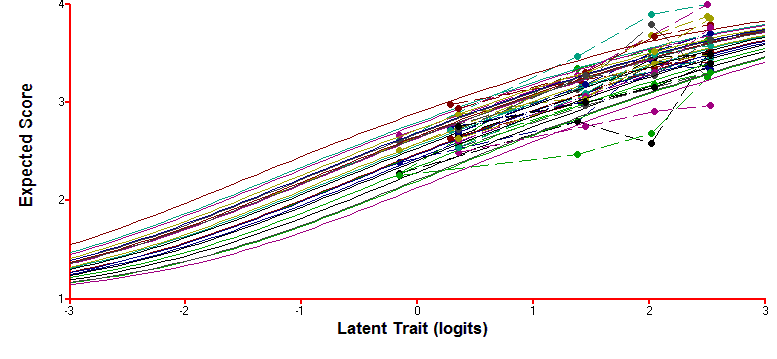


*Figure A27.* Community data set multidimensional RACES ESCs. The x-axis indicates the expected response (i.e., 1 = “Strongly Disagree”, 2 = “Disagree”, 3 = “Agree”, and 4 = “Strongly Agree”) predicted by the model. The y-axis indicates the level of the latent trait. Each individual line represents the ESC of one item. The smooth lines represent the expected score, given the trait level of an individual. The broken lines represent the actual data. The left most ESCs are the items that will be endorsed by individuals low to high on the latent trait. The right most ESCs are the items that will only be endorsed by individuals high on the latent trait.
